# Ecological amplitude and indication potential of mining bees (*Andrena* spp.): a case study from the post-agricultural area of the Kampinos National Park (Poland)

**DOI:** 10.1038/s41598-024-59138-9

**Published:** 2024-04-28

**Authors:** Katarzyna Szczepko-Morawiec, Bogdan Wiśniowski, Ewelina Motyka, Waldemar Celary, Andrzej Kruk

**Affiliations:** 1 Department of Biodiversity Studies, Didactics and Bioeducation, Faculty of Biology and Environmental Protection, University of Lodz, Lodz, Poland; 2 Institute of Agricultural Sciences, Land Management and Environmental Protection, University of Rzeszów, Rzeszów, Poland; 3 Bukowiec, Poland; 4 Institute of Biology, The Jan Kochanowski University, Kielce, Poland; 5 Department of Ecology and Vertebrate Zoology, Faculty of Biology and Environmental Protection, University of Lodz, Lodz, Poland

**Keywords:** Biodiversity, Conservation biology, Ecology, Zoology

## Abstract

The mining bee (*Andrena* spp.) play a key role in ensuring plant and animal diversity. The present study examines their diversity in a post-agricultural landscape exemplified by the Kampinos National Park (KNP), a UNESCO Biosphere Reserve in Poland. The following hypotheses were addressed: (H1) the mining bees demonstrate a narrow ecological amplitude, (H2) there are no indicator species for particular habitats, and (H3) the studied mining bees have the same ecological preferences to those presented in the literature. A total of 40 catch per unit effort samples (CPUE) were collected across various habitats with different soil humidity. Forty-six species were recorded, representing 46% of mining bees and approximately 10% of the known Polish bee fauna. Nineteen of the recorded species (41%) were assigned to CR-NT threat categories, indicating that the national park plays a significant role in preserving mining bee species diversity and their conservation. None of the hypotheses (H1, H2, H3) were confirmed. The mining bees were found to demonstrate a wide ecological amplitude. Surprisingly, habitats located in dry and wet soils were both characterised by high abundance and species richness. Seventeen indicators were distinguished among the dominant and rarer species. Our findings suggest that *Andrena nigroaenea* and *A*.* ventralis* (lower humidity), as well as *A*.* alfkenella* and *A*.* minutuloides* (higher humidity), have different significant relationships with habitat soil humidity to those reported in the literature.

## Introduction

The bees (Apoidea, Anthophila) are a monophyletic group with approximately 20,000 species described worldwide^1^. The genus *Andrena* Fabr. (family Andrenidae), comprising short-tongued, solitary bees ranging in length from 6 mm to almost 20 mm^1^, is one of the largest of all bee genera, with more than 1500 described species in the world fauna^1,2^, and about 400 in Europe^3^. It comprises more than 20% of the bees of Poland, with 100 of the currently-known species in the country^4^. *Andrena* is distributed throughout North and Central America, North Africa and Eurasia, including the Far East; the group is absent from South America, Australia and Oceania^1^.

Wild bees, including *Andrena* species, are the main plant pollinators in many ecosystems^5^. As they are needed for the pollination of many entomophilous species and the successful commercial production of fruits and vegetables^6^, they are often considered keystone species^7^. Some species are oligolectic, i.e. they collect nectar and pollen from a single plant family, and sometimes only a single genus or species. Most andrenids are early spring species; however, some have later flight periods, and some spring species have a second generation in late summer^8^. They emerge when relatively few pollinators are active, making them very important pollinators of early blooming plants, as well as commercially-grown apples, blueberries, strawberries and a few other early-flowering crops^6^. Furthermore, wild bee pollinators improve the fruits of crops and their quality, regardless of the abundance of honeybees^9^.

Some *Andrena* species have been awarded the common name *mining bees* or *miner bees*, due to their nesting preferences. The females dig nests in soil, mostly in areas with bare or sparse vegetation^2^. The nests are provisioned with pollen and/or nectar^8^, and then the cells are closed, and larvae develop while feeding on the stored food. Some mining bees show a clear preference for a particular habitat such as sandy areas, forest edges, or midforest clearings^2,10^.

Ecological studies of this group of bees have previously been concerned mainly with descriptions of andrenid assemblages in various plant communities and ecosystems; they therefore lack analyses of the environmental factors determining their diversity. Also, andrenids have been treated as indicators of biodiversity e.g.^11,12^. Other studies have regarded them as a component of Aculeata or Apoidea assemblages occurring in certain habitats, such as fallows^13^, meadows^14^, forests^15,16^, limestone, sand quarries and mines^17^, clay and sand pits^18^, off-road motorcycle circuits^19^, urban, suburban and nonurban zones under different degree of human pressure^20^, urban parks and forests^21^ and agricultural landscapes^22^. Both semi-natural (man-made) and natural habitats have been studied in national parks such as the Kampinos National Park, Białowieża National Park, and Wielkopolska National Park in Poland, as well as the Pinnacles National Park in California (USA)^10,23^.

Bees require three key factors to thrive: food resources, the availability of suitable microhabitats protected from unfavourable biotic (predators, parasitoids) and abiotic (moisture, rain, drought) factors, and the availability of space and material for nest construction^8^. Bee populations have decreased significantly in recent decades^24^. While multiple causes have been identified, the most influential are believed to be loss of floral resources and the reduced availability or suitability of open areas resulting from urbanisation, agricultural intensification or afforestation^15,25–27^.

Throughout the temperate zone of Central Europe, including Poland, the natural succession of plant communities results in the establishment of forested areas. In natural conditions, only a small area in any forest consists of open ground, and its distribution changes quite rapidly over time as a result of vegetative succession^28^. Human activity causes disturbances in the natural environment; while this may lead to the disappearance of suitable habitats, it may also produce many new ones, which may have a positive influence on the biodiversity of bees, including mining bees of the genus *Andrena*^8,27^.

Wild bees are considered endangered in Europe. According to the *European Red List of Bees*^29^*,* 0.4% of species are Critically Endangered, 2.4% are Endangered, and 1.2% Vulnerable. Another 5.2% are classified as Near Threatened. Furthermore, for more than half (56.7%) of the species in Europe, insufficient data exists to evaluate their risk of extinction: these have hence been classified in the Red List as *Data Deficient*. As more data becomes available, many of these species may also prove to be threatened. Among these, ground-nesting species are particularly endangered, due to the environmental changes associated with vegetation succession^26,30^. For example, in Belgium, ground-nesting bees are more threatened (32.5%) than those nesting in existing cavities above ground (23.6%)^31^. In Europe, studies suggest that among the entire Anthophila group, only 34.2% of species appear to be nonthreatened^29^, ranging from 41.7% of the family Megachilidae to only 23.3% of the Andrenidae. In the subfamily Andreninae, represented in Europe mainly by bees of the genus *Andrena*, this percentage is even lower, i.e. 22.3%. Thus, it can be concluded that the Andreninae, including in particular the genus *Andrena*, is the most endangered European bee taxon.

Many wild bee species in Poland are considered rare and only occur locally^32^. The *Red List of Vanishing and Endangered Animals in Poland* in 2002 included half of the species recorded in the country^33^, with most of these records assessed as Data Deficient. The list of the genus *Andrena* was more preliminary in character, because no species was listed in the categories EX/RE (extinct/regionally extinct) or CR (critically endangered), although some taxa were not recorded in Poland for a relatively long period of time^34^. According to the verified Red List of the genus *Andrena*^10^, over 87% of the species that occur in the country are more or less endangered. An updated Red List of threatened wild bees in Poland is clearly necessary. In selected *Andrena* species, verification of their threat status has mostly led to the reclassification of the species into higher categories of threat^10,34^ compared to the previous Red List^33^.

To successfully conserve andrenid species, it is first necessary to understand their habitat preferences. Hence, the present study examines the ecology of andrenids in the Kampinos National Park (KNP), Central Poland, an eminently suitable site for this purpose. This area was selected for three main reasons: (1) the availability of the study area, (2) national parks play an important role in biodiversity protection, (3) the KNP is characterised by changes in habitats due to natural succession. The KNP itself is a UNESCO Biosphere Reserve. It has a number of open areas, which naturally arise as a result of processes that temporarily destroy tree cover, such as fires, windfalls, or outbreaks of folivorous insects, or as a consequence of human activities^35,36^. The latter has been the most significant factor shaping the KNP. Its territory has been managed consistently in a varied manner (hay-making, cattle grazing, agriculture), which provides a mosaic of habitats and ensures suitable conditions for many bee species^37,38^. However, continued abandonment of traditional management leads to the development of forest communities in formerly open areas, which can threaten various hymenopteran groups, such as pompilid, chrysidid, and vespid wasps^39–41^.

The conducted research was based on the following hypotheses: (H1) the mining bees demonstrate a narrow ecological amplitude, (H2) there are no indicator species for particular habitats, and (H3) the studied mining bees have the same ecological preferences to those presented in the literature.

## Study area

The Kampinos National Park (KNP) (52° 25′–52° 15′ 30″ N; 20° 17′–20° 53′ E) is located on the Mazovian Lowland in Central Poland. It is one of two national parks in Europe and of three in the world directly adjacent to the capital of the country. The park was created in 1959 to protect the unique complex of inland dunes and wetland areas, natural forest communities and rich fauna^35^. In 2000, the park was declared a UNESCO World Biosphere Reserve, “Puszcza Kampinoska”, and since 2004, it has also been part of the Nature 2000 network (site 'Puszcza Kampinoska' PLC 140,001)^42^. More than 73% of the Park is covered with forests. Infertile dune lands are covered with fresh coniferous forest, usually inhabited by pine and silver birch. Forest communities are dominated by mixed pine forests, mostly *Querco roboris-Pinetum*, occurring together with *Peucedano-Pinetum* or *Molinio-Pinetum* pine forests on wetter habitats, *Fraxino-Alnetum* alder-ash riparian forests occurring along watercourses and on the edges of *Ribo nigri-Alnetum* swamp alder forests, *Tilio-Carpinetum* oak-hornbeam forests covering elevations among wetlands, and small patches of *Potentillo albae-Quercetum* thermophilous oak forests on some slopes of dunes^35^.

**Figure 1:**
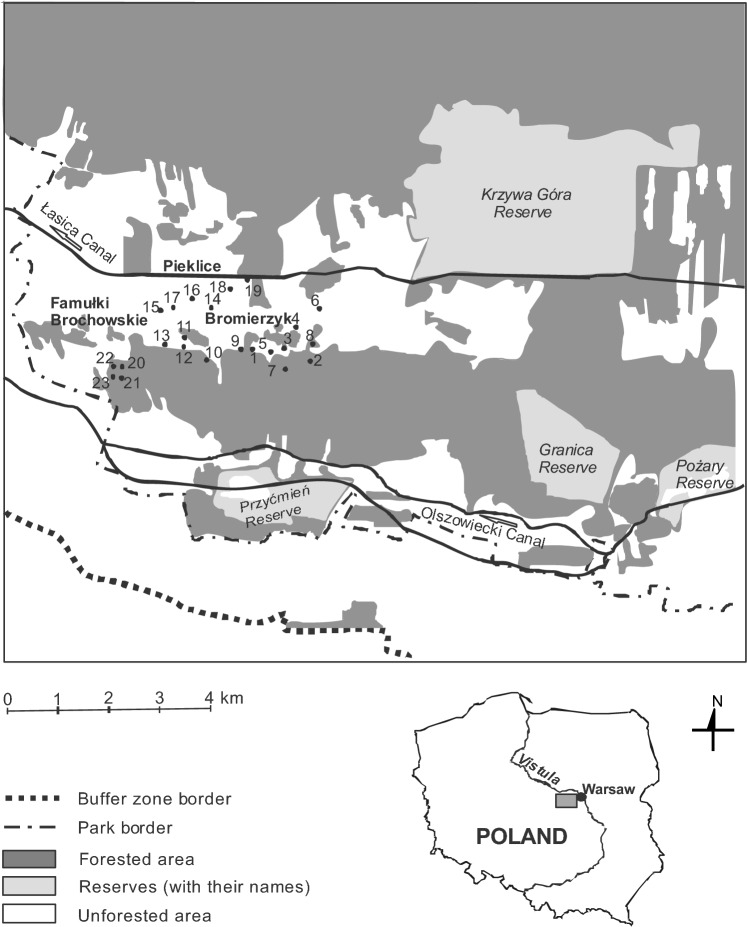
Study area with sampling sites marked with black circles. The names of the localities are given in bold.

The KNP covers 38,544 ha, including 4636 ha of strict protection areas (12% of the Park) and 37,756 ha of a buffer zone. The park has a belt-like structure consisting of wide belts of swampy depressions (the Łasica Canal depression and bipartite southern belt of the Olszowiecki and Zaborów Canals) separated by belts of sand dunes running parallel to the Vistula River, i.e. from east to west (Fig. 1). The swampy belts are covered by meadows, reed beds, willow shrubberies, alder-ash and alder forests. The sand dunes are among the best preserved inland dunes in Europe. They are mainly covered by woodland, mainly pine forests^35^.

The area of the current park has been depopulated and incorporated into the KNP by degrees since the late 1970s. During this period, the land was gradually forested or left to natural succession, and as such, its landscape is highly heterogeneous. Natural (forests) habitats are interspersed with semi-natural ones (grasslands, dunes, meadows, fallow fields), and both abandoned and inhabited human settlements (buildings and/or farms)^35^.

The Kampinos National Park is located in the temperate zone of moderate mean latitudes. In this area, six seasons can be distinguished during the year, among which the longest is winter, with an mean duration of 101 days. The growing season, i.e. with temperatures exceeding 5 °C, lasts approximately 185 days a year. The mean annual air temperature is 7.7 °C, which is 1.1 °C lower than that of neighbouring areas. A high number of days are characterised by ground frost: a mean number of 38.6 in the summer half of the year. The mean total annual precipitation is around 550 mm, with precipitation occurring on a mean number of 124.5 days per year. The distribution of rainfall in the KNP is distinctly uneven, with lower total amounts noted in the west and higher amounts in the central and eastern parts. Westerly winds prevail^35^.

This study was carried out at 23 sites in the western part of the KNP (Fig. 1, Table 1).

**Table 1:** Types of sampled habitats (compare with Table 2).

Habitat type	Number of sites
Abandoned farm with the remnants of foundations and walls, in a ruderal habitat (*Artemisietea* class)	1
Fresh coniferous forest *Querco roboris-Pinetum*	1
Degenerate alder swamp forest *Ribo nigri-Alnetum*	1
Old pear and apple trees in a ruderal habitat (*Artemisietea* class)	1
A sand dune, area 150 m^2^ and height 1.3 m, surrounded by *Querco roboris-Pinetum* coniferous forest	1
Psammophilous grasslands of the *Koelerio glaucae-Corynephoretea canescentis* class, in a small grassland area (150 m^2^) surrounded by a woodland composed of oak *Quercus robur*, pine *Pinus sylvestris* and black locust *Robinia pseudoacacia*	1
Psammophilous grasslands of the *Koelerio glaucae-Corynephoretea canescentis* class, in a grassland area (450 m^2^) surrounded by *Cladonia* heath, *Pinus sylvestris* forest, *Betula pendula* scrub and *Robinia pseudacacia* woodland	1
A wet meadow of the *Calthion* alliance (not mowed)	1
A fresh meadow of the *Arrhenatherion* alliance (mowed once a year in June)	1
Fallow (3000–4000 m^2^) left to natural succession, the last crops being cereals or potatoes	14

## Materials and methods

The samples were collected in 2002–2006, between early April and the beginning of October. Each sample was assigned a code consisting of (i) site number (two digits), (ii) two letters indicating the habitat sampled (AF—abandoned farm, FA—fallow, FR—forest, FT—fruit trees, ME—meadow, PG—psammophilous grassland, SD—sand dune), (iii) two digits indicating the year of sample collection and (iv) a letter for the level of soil humidity (for open areas only): D—autogenic (dry), S—semihydrogenic, H—hydrogenic (humid) (Tables 2, 3). For example: the sample 21FA06D was collected in 2006 at site No 21 located in a fallow on autogenic soils.

**Table 2:** Basic information on samples and sampling sites.

Sample code	Site number	Sampled habitat	Fallow age	Year of sampling	Number of *Andrena* species
01FT02S	1	Fruit trees	×	2002	15
02PG02D	2	Psammophilous grassland	×	2002	6
03SD02D	3	Sand dune	×	2002	8
04ME02H	4	Meadow	×	2002	10
05PG02D	5	Psammophilous grassland	×	2002	8
06AF02H	6	Abandoned farm	×	2002	14
07FR02D	7	Coniferous forest	×	2002	14
07FR03D	7	Coniferous forest	×	2003	8
08FR02H	8	Alder forest	×	2002	6
09FA02S	9	Fallow	2	2002	1
09FA03S	9	Fallow	3	2003	11
09FA04S	9	Fallow	4	2004	19
09FA05S	9	Fallow	5	2005	14
10FA03D	10	Fallow	10	2003	13
10FA04D	10	Fallow	11	2004	19
11FA03S	11	Fallow	4	2003	10
11FA04S	11	Fallow	5	2004	12
11FA05S	11	Fallow	6	2005	14
12FA03S	12	Fallow	3	2003	11
12FA04S	12	Fallow	4	2004	13
13FA03S	13	Fallow	7	2003	6
13FA04S	13	Fallow	8	2004	11
14FA03H	14	Fallow	1	2003	17
14FA04H	14	Fallow	2	2004	17
15ME03H	15	Meadow	×	2003	4
15ME04H	15	Meadow	×	2004	15
16FA04H	16	Fallow	1	2004	14
16FA05H	16	Fallow	2	2005	14
16FA06H	16	Fallow	3	2006	14
17FA04H	17	Fallow	2	2004	14
18FA04H	18	Fallow	1	2004	15
18FA05H	18	Fallow	2	2005	16
19FA04H	19	Fallow	1	2004	17
20FA05D	20	Fallow	10	2005	16
21FA05D	21	fallow	15	2005	9
21FA06D	21	Fallow	16	2006	9
22FA05D	22	Fallow	5	2005	15
22FA06D	22	Fallow	6	2006	11
23FA05D	23	Fallow	20	2005	13
23FA06D	23	Fallow	21	2006	17

The code for each mining bee sample consists of the site number, letters indicating the habitat (*AF* abandoned farm, *FA* fallow field, *FR* forest, *FT* fruit trees, *ME* meadow, *PG* psammophilous grassland, *SD* sand dune), followed by two digits indicating the year the sample was collected and a letter for soil humidity level: *D* dry (autogenic), *S* semihumid (semihydrogenic), *H* humid (hydrogenic).

**Table 3:** Number of mining bee samples assigned to SOM subclusters in relation to the type of habitat and humidity of soil in open areas.

Subcluster	Open habitas								FR	Total
	Autogenic			Semihydrogenic		Hydrogenic				
	FA	PG	SD	FA	FT	FA	ME	AF		
X1	3	2	1	9		1	1		1	18
X2					1		1	1	2	5
Y1				2		8	1			11
Y2	6									6
Total	9	2	1	11	1	9	3	1	3	40

*AF* abandoned farm, *FA* fallow field, *FR* forest, *FT* fruit trees, *ME* meadow, *PG* psammophilous grassland, *SD* sand dune.

Samples were planned to be collected from each site 2–3 times, i.e. in 2–3 successive years (Table 2). However, this was not always possible, especially since our research included only "full" samples covering the cycle from the beginning of April to the beginning of October. The reason for the inability to collect samples was (1) destruction or theft of traps, which eliminated the result for a given year, (2) destruction of the habitat, e.g. as a result of mowing the meadow, its flooding, plowing the field or planting it with tree seedlings, (3) entry ban due to the decision of the farmer or the KNP administration due to forest maintenance treatments, cutting trees or removal of wood. We decided to include the sites sampled in a single year in the study because they increased the variation observed in the dataset and thus allowed a broader perspective in the analysis of preferences of *Andrena* species.

The sampling methods were standardized. Catching was carried out per unit effort (CPUE) using water-filled pan traps (coloured bowls, 20 cm in diameter, filled with soapy water); these are regarded as a standard and effective technique for collecting flying insects, including bees, in open and forest habitats^11,16^. The traps were two-thirds filled with a mixture of water (95%), glycol (5%) as a preservative, and a detergent to break the surface tension. At each site, three traps (two yellow and one white) were used. Depending on the type of site, they were either hung on trees, placed on the ground, or hung on poles at a height similar to the mean height of the surrounding vegetation. Each trap was emptied every 10 days, 19 times in a given season. The 19 aggregated catches from three traps at a site were treated as a sample.

In the field, the bees were preserved in 75% ethanol. Following this, in the laboratory, they were mounted, labelled, and deposited in the Department of Biodiversity Studies, Didactics and Bioeducation of the University of Lodz. Their identification was based on Amiet et al.^43^, Dylewska^44^, Schmid-Egger and Scheuchl^45^.

The bees were classified according to nomenclature of the Fauna Europaea^3^. IUCN threat categories were adopted according to Głowaciński^46^. The Polish Red List of Bees was published in 2002^33^ as part of the *Red list of threatened animals in Poland*^46^, and repeated unchanged in 2004^32^. The list included 42 species of the 93 known at that time from Poland. The published lists were unreliable, because no species were listed in either categories EX/EX? nor CR, although some species were not recorded in Poland for a fairly long period of time. The species identified during the present study were characterized in terms of their threat status in Poland according to IUCN criteria, based on the revised list of threatened *Andrena* species from Poland^10^; the list includes the status of 95 species of this genus, including seven species evaluated as probably extinct (EX? category of threat), 12 critically endangered (CR), nine endangered (EN), 15 vulnerable (VU), 20 near threatened (NT), 16 least concern (LC), and four data deficient (DD). In addition, 12 species were evaluated as unthreatened in Poland (category UT proposed by Motyka^10^).

Based on their diet specialization, the mining bee species were classified as either oligolectic, i.e. collecting pollen from several closely-related species or genera of a single family of flowering plants, and polylectic, i.e. collecting pollen from a wide range of flowering plant species. No monolectic species were identified in the collected material. The specimens were also grouped according to their environmental preferences: (a) eurytopic, occurring from lowlands to high mountains, both in open and forested areas, (b) polytopic, inhabiting a wide range of habitats, but preferring either open or forested areas, and (c) oligotopic, associated with a particular type of habitat, mostly dry and on sand, or of steppe character. The bees were also classified into four groups based on their nesting preferences connected with soil moisture: (a) dry, (b) dry and moderately moist; (c) moderately moist, (d) moderately moist and moist. They were also classified into two groups according to the beginning of the foraging season: (a) early spring and, (b) late spring. Only one summer species was recorded in the collected material, and this was included in the late spring group for the sake of the analysis. The above traits for each bee species were collected from literature^43,45,47,48^.

A Kohonen artificial neural network (ANN), also referred to as a self-organizing map (SOM), was used to recognize patterns in the abundances of mining bee species. ANNs are simple structural and functional models of a human brain. They consist of processing units called neurons. They do not require any a priori specification of the model underlying a studied phenomenon because they can learn it based on the processed data^49^. ANNs easily deal with wild organism counts which are non-linearly related and often have a skewed distribution (because of many zeroes).

A few studies have previously employed Kohonen ANNs to examine hymenopterans including ants^50^, polistinae wasps^51^, spider, cuckoo and vespid wasps^39–41^. However, the present study is the first to use Kohonen ANNs to examine patterns in mining bee (andrenid) assemblages.

**Figure 2:**
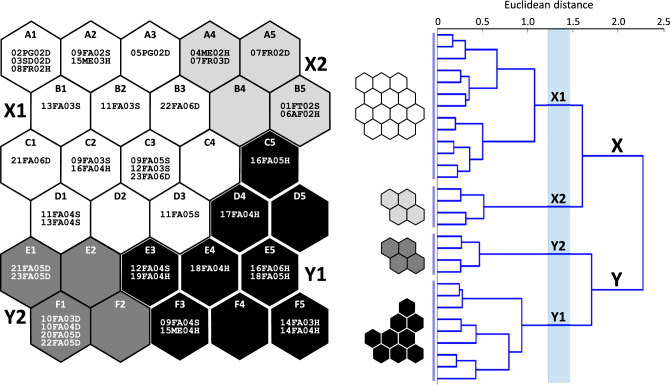
Forty samples of mining bees in 30 SOM output neurons, which are arranged in a two-dimensional grid (6 × 5). Clusters (X and Y) and subclusters (X1, X2, Y1 and Y2; shaded) of neurons and respective sample models were determined by hierarchical cluster analysis. The code for each sample consists of the site number and two letters for the habitat (AF—abandoned farm, FA—fallow, FT—fruit trees, FR—forest, ME—meadow, PG—psammophilous grassland, SD—sand dune); each code ends with two digits indicating the year the sample was collected, and a letter for the level of soil humidity: D—autogenic (dry), S—semihydrogenic, H—hydrogenic (humid).

Kohonen ANNs are built of two layers of neurons (input and output). The number of input neurons was equal to the number of input variables (i.e. the abundances of 46 species of mining bees) because each log-transformed variable was received by a single input neuron. The output layer consisted of 30 neurons that were arranged on a two-dimensional 6 × 5 grid (Fig. 2); the grid size was determined based on the heuristic principle that the number of output neurons should be close to 5√n, where n in this case is number of mining bee samples, i.e. n = 40 (result: 32; see^52^). Each input neuron was connected to all output neurons, and repeatedly transmitted information to them during the training process. The input neurons had no further significance for pattern recognition^53^.

Based on strengthened or weakened intensity (weight) of the received signals, a model of a virtual mining bee sample (MBS) was created in each output neuron. The similarity of the sample models was related to the topology of the SOM, i.e., the virtual MBSs in distant neurons differed more from each other than in the neighbouring neurons. Following this, the clusters of the virtual MBSs, and thus the respective output neurons, were identified by hierarchical cluster analysis based on the Ward algorithm and Euclidean distance measure^52,54^.

Finally, each real MBS was assigned to the best matching virtual MBS and the respective output neuron. When a respective virtual MBS was not the best match for any real MBS, the given output neuron remained ‘empty’, i.e. without any real MBS assigned (although with a virtual MBS). However, if the given virtual MBS was the best match for more than one real MBS, the respective output neuron contained two or more real MBSs. Thus, the Kohonen ANN progressively recognized patterns in mining bee assemblages, distinguished classes of virtual MBSs, and assigned real MBSs to them.

A batch training algorithm was chosen to train the network, because it does not require any training rate factor to be specified^55^. The network training procedure was performed using the SOM Toolbox^56^, developed in the Laboratory of Information and Computer Science at the Helsinki University of Technology (http://www.cis.hut.fi/projects/somtoolbox/).

The SOM Toolbox allows the associations between mining bee species and SOM regions to be visualised in the form of greyness gradients over a two-dimensional grid^49^. This visualization may be very helpful in formulating ecological conclusions, as species with the same greyness pattern in the SOM usually have similar habitat preferences. However, as the SOM Toolbox does not provide any statistical verification of those associations, the untransformed mining bee abundance data were also subjected to Indicator Species Analysis (ISA), which is based on indicator values (IndVals)^57^.

The IndVal (range 0–100%) of the mining bee species *i* in the (sub)cluster *j* is a product of three values: (1) A_ij_—the mean abundance of the species *i* in real MBSs assigned to the subcluster *j*, divided by the sum of its average abundances in all subclusters (%), (2) F_ij_—the constancy of occurrence of the species *i* (%) in real MBSs assigned to the subcluster *j*, and (3) the constant 100, used to obtain the percentages as follows:

$${\text{IndVal}}_{ij} = {\text{A}}_{ij} \times {\text{F}}_{ij} \times {1}00$$

A_*ij*_ = abundance_*ij*_/abundance_*i*._, F_*ij*_ = N real samples_*ij*_/N real samples_.*j*_

The maximum IndVal (100%) was observed when all MBSs with the species* i* were assigned to a single subcluster of output neurons, and when the species *i* was recorded in all MBSs assigned to that subcluster^57^.

The significance level of the maximum observed IndVal for each species was calculated using a Monte Carlo test. Hence, the IndVals and SOM species planes express both numerically and respectively, in the form of a gradient greyness, the significance of each subcluster of output neurons to a species. As such, the values complement each other: both enable identification of the subclusters of neurons in which a given species is most frequent and abundant, and hence the abiotic conditions it prefers.

At each sampling site, the percentage of bare ground site was determined and ranked as either 1 (0–25% of exposed surface), 2 (26–50%), 3 (51–75%) or 4 (76–100%). Similarly, the soil was classified into one of the three following types: autogenic (dry), i.e. podzolized soil or podzol; semihydrogenic, i.e. brown soils or black earth; hydrogenic (humid), i.e. moorsh or muckous soil. Soil humidity was ranked as 1—autogenic (dry), 2—semi-hydrogenic or 3—hydrogenic (humid) soil. The soil type was determined in ArcGIS ver. 9.3.1 software by superimposing geographic GPS coordinates (Garmin GPSMap, 60Cx) of sites on GIS soil maps^58^. This analysis was supplemented with descriptive information on soil types^35,59^.

Using the Kruskal–Wallis test and the post hoc Dunn test^60,61^, the SOM subclusters were compared according to the following four dimensions: total abundance, number of mining bee species, availability of bare land and soil humidity.

## Results

During the study, 40 samples were collected. A total of 4335 mining bee individuals belonging to 46 species were recorded. Among them: (1) one species had the category CR (*Andrena gallica*), (2) one had the category EN (*Andrena symphyti*), (3) seven species had the category VU, (4) 10 species had the category NT, (5) 13 species had the category LC, and 6) two species had the category DD; 12 species were defined as unthreatened (category UT; all according to Motyka^10^) (Tables 4, 5 and 6).

**Table 4:** Relative abundance (A), relative frequency (F) and IndVals (I) (all in %) and total observed abundance (TA, in number of specimens) of mining bees divided into two groups: species recorded in ≥ 3 samples (α) and remaining species (β).

SOM subcluster		Species α																
		**2.b**. *A*.* alfkenella* Perkins, 1914 ^**NT**^	**2.d.** *A*.* apicata* Smith, 1847 ^**NT**^	**3.d.** *A*.* barbilabris* (Kirby, 1802) ^**LC**^	**3.a.** *A*.* bimaculata* (Kirby, 1802) ^**NT**^	**2.c.** *A*.* chrysosceles* (Kirby, 1802) ^**LC**^	**2.b.** *A*.* cineraria* (Linnaeus, 1758) ^**LC**^	**2.c.** *A*.* clarkella* (Kirby, 1802) ^**LC**^	**2.b.** *A*.* dorsata* (Kirby, 1802)	**2.b.** *A*.* falsifica* Perkins, 1915 ^**NT**^	**1.b.** *A*.* flavipes* Panzer, 1799	**3.a.** *A*.* florivaga* Eversmann, 1852 ^**DD**^	**3.b.** *A*.* fucata* Panzer, 1798 ^**NT**^	**2.b.** *A*.* fulva* (Müller, 1766) ^**LC**^	**2.b.** *A*.* fulvago* (Christ, 1791) ^**NT**^	**3.c.** A. *fulvida* Schenck*,* 1853 ^**VU**^	**1.c.** *A*.* gravida* Imhoff, 1832 ^**LC**^	**1.c.** *A*.* haemorrhoa* (Fabricius, 1781)
	TA	7	19	168	24	12	551	5	37	33	191	8	16	61	10	3	5	988
X1	A	0	9	52	0	8	6	0	8	3	12	38	6	6	44	36	0	3
	F	0	17	94	0	6	72	0	22	6	61	17	11	17	22	11	0	67
	I	0	2	50	0	0	5	0	2	0	7	6	1	1	10	4	0	2
X2	A	0	0	15	39	30	3	77	6	0	2	0	68	78	0	64	0	31
	F	0	0	60	20	20	80	40	20	0	20	0	40	80	0	20	0	100
	I	0	0	9	8	6	2	**31**	1	0	0	0	27	**62**	0	13	0	31
Y1	A	100	72	5	4	48	29	23	68	76	70	62	0	13	0	0	69	57
	F	36	45	45	18	27	100	9	82	82	100	27	0	55	0	0	36	100
	I	**36**	33	2	1	13	29	2	**56**	**62**	**70**	17	0	7	0	0	25	57
Y2	A	0	19	28	57	13	61	0	19	21	16	0	25	4	56	0	31	8
	F	0	33	100	33	17	100	0	50	33	83	0	33	17	33	0	17	83
	I	0	6	28	19	2	**61**	0	9	7	13	0	8	1	19	0	5	7

SOM subcluster		Species α																
		**2.c.** *A*.* helvola* (Linnaeus, 1758) ^**LC**^	**2.b.** *A*.* labiata* Fabricius, 1781 ^**NT**^	**3.c.** *A*.* lapponica* Zetterstedt, 1838 ^**LC**^	**1.a.** *A*.* minutula* (Kirby, 1802)	**1.a.** *A*.* minutoloides* Perkins, 1914	**3.d.** *A*.* mitis* Schmiedeknecht, 1883 ^**NT**^	**2.d.** *A*.* nigroaenea* (Kirby, 1802)	**1.c.** *A*.* nitida* (Müller, 1776)	**2.b.** *A*.* ovatula* (Kirby, 1802) ^**LC**^	**3.a.** *A*.* pilipes* Fabricius, 1781 ^**LC**^	**2.a.** *A*.* praecox* (Scopoli, 1763)	**1.b.** *A*.* subopaca* Nylander, 1848	**2.b.** *A*.* tibialis* (Kirby, 1802) ^**LC**^	**2.d.** *A*.* vaga* Panzer, 1799	**2.b.*** A*. *varians* (Rossi, 1791) ^**NT**^	**2.d.** *A*.* ventralis* Imhoff, 1832	**2.b.** *A*.* viridescens* Viereck, 1916 ^**VU**^
	TA	32	34	15	11	33	17	485	77	32	28	53	50	36	546	3	720	6
X1	A	11	34	20	16	4	7	13	16	1	0	6	17	10	7	0	12	23
	F	22	44	11	11	6	11	72	44	6	0	33	33	22	83	0	89	11
	I	2	15	2	2	0	1	9	7	0	0	2	6	2	6	0	10	3
X2	A	61	0	62	29	0	52	2	8	0	0	66	64	6	22	81	3	0
	F	60	0	40	20	0	40	80	60	0	0	100	80	20	100	40	40	0
	I	37	0	25	6	0	21	2	5	0	0	**66**	**51**	1	22	**33**	1	0
Y1	A	6	59	0	7	96	20	15	38	0	8	19	12	74	40	19	8	77
	F	18	55	0	9	45	36	82	100	0	18	64	27	64	100	9	91	27
	I	1	32	0	1	**44**	7	12	38	0	2	12	3	**47**	40	2	8	21
Y2	A	22	6	17	48	0	22	70	38	99	92	9	8	10	30	0	77	0
	F	17	17	17	17	0	50	100	83	100	100	17	33	17	83	0	100	0
	I	4	1	3	8	0	11	**70**	32	**99**	**92**	1	3	2	25	0	**77**	0

SOM subcluster		Species β																
		**1.c.** *A*.* bicolor* Fabricius, 1775	**3.a.** *A*.* chrysopyga* Schenck, 1853 ^**VU**^	**2.c.** *A*.* denticulata* (Kirby, 1802) ^**NT**^	**3.b.** *A*.* gallica* Schmiedeknecht, 1883 ^**CR**^	**3.a.** *A*.* gelriae* Van der Vecht, 1927 ^**VU**^	**2.c.** *A*.* humilis* Imhoff, 1832 ^**LC**^	**3.a.** *A*.* limata* Smith, 1853 ^**VU**^	**3.b.** *A*.* nycthemera* Imhoff, 1868 ^**VU**^	**2.a.** *A*.* propinqua* Schenck, 1853 ^**DD**^	**3.a.** *A*.* ruficrus* Nylander, 1848 ^**VU**^	**3.d.** *A*.* symphyti* Schmiedeknecht, 1883 ^**EN**^	**2.b.** *A*.* wilkella* (Kirby, 1802) ^**LC**^					
	TA	1	1	1	1	2	3	1	1	2	2	1	3					
X1	A	100	0	0	0	100	55	0	0	38	100	100	0					
	F	6	0	0	0	6	6	0	0	6	11	6	0					
	I	6	0	0	0	6	3	0	0	2	11	6	0					
X2	A	0	0	100	0	0	0	0	100	0	0	0	0					
	F	0	0	20	0	0	0	0	20	0	0	0	0					
	I	0	0	20	0	0	0	0	20	0	0	0	0					
Y1	A	0	100	0	100	0	45	100	0	62	0	0	0					
	F	0	9	0	9	0	9	9	0	9	0	0	0					
	I	0	9	0	9	0	4	9	0	6	0	0	0					
Y2	A	0	0	0	0	0	0	0	0	0	0	0	100					
	F	0	0	0	0	0	0	0	0	0	0	0	33					
	I	0	0	0	0	0	0	0	0	0	0	0	**33**					

IndVals highest in a given SOM subcluster at *p* ≤ 0.05 are bolded and underlined (significance levels are presented in Fig. 4). Species with A = 100% were recorded exclusively in the samples assigned to a given SOM subcluster. Information on: habitat preferences is marked by number preceding species name (1—eurytopic, occurring from lowlands to high mountains, both in open and forested areas; 2—polytopic, inhabiting wide range of habitats but preferring either open or forested areas; 3—oligotopic, associated with particular types of habitats, mostly dry and on sand or of steppe character), nesting preferences connected with soil moisture is marked by the letter preceding the species name (a—dry, b—dry and moderately moist, c—moderately moist, d—moderately moist and moist). Categories of threat according to verified list of threatened species^10,34^: CR—critically endangered, EN—endangered, VU—vulnerable, NT—near threatened, LC—least concern, DD—data deficient, remaining species—not threatened.

**Table 5:** Rare species (groups β from Table 4) with threat categories NT-CR (*CR* critically endangered, *EN* endangered, *VU* vulnerable, *NT* near threatened).

No.	*Andrena* species	Threat category	Soil humidity	Site	SOM subcluster
1	*A*.* chrysopyga*	VU	H	16FA05H	Y1
2	*A*.* denticulata*	NT	D	07FR03D	X2
3	*A*.* gallica*	CR	S	09FA04S	Y1
4	*A*.* gelriae*	VU	D	22FA06D	X1
5	*A*.* limata*	VU	H	17FA04H	Y1
6	*A*.* nycthemera*	VU	H	06AF02H	X2
7	*A*.* ruficrus*	VU	S	09FA05S, 12FA03S	X1
8	*A*.* symphyti*	EN	D	23FA06D	X1

**Table 6:** Threat categories of *Andrena* species recorded in Kampinos National Park according to various authors (*CR* critically endangered, *EN* endangered, *VU* vulnerable, *NT* near threatened, *LC* least concern, *UT* unthreatened, *DD* data deficient).

No	*Andrena* species	Categories of threat according to		
		Banaszak^32,33^ (Poland)	Nieto et al.^29^ (Europe)	Motyka^10^, Wiśniowski et al.^34^ (Poland)
1	*A*.* alfkenella*	VU	DD	NT
2	*A*.* apicata*		DD	NT
3	*A*.* barbilabris*		DD	LC
4	*A*.* bicolor*		LC	UT
5	*A*.* bimaculata*	DD	DD	NT
6	*A*.* chrysopyga*		DD	VU
7	*A*.* chrysosceles*		DD	LC
8	*A*.* cineraria*		LC	LC
9	*A*.* clarkella*		DD	LC
10	*A*.* denticulata*		DD	NT
11	*A*. *dorsata*		DD	UT
12	*A*.* falsifica*	VU	DD	NT
13	*A*.* flavipes*		LC	UT
14	*A*.* florivaga*		NT	DD
15	*A*.* fucata*		DD	NT
16	*A*.* fulva*		DD	LC
17	*A*.* fulvida*	VU	NT	VU
18	*A*.* fulvago*		DD	NT
19	*A*.* gallica*		LC	CR
20	*A*.* gelriae*		DD	VU
21	*A*.* gravida*		DD	LC
22	*A*.* haemorrhoa*		LC	UT
23	*A*.* helvola*		DD	LC
24	*A*.* humilis*		DD	LC
25	*A*.* labiata*		DD	NT
26	*A*.* lapponica*		LC	LC
27	*A*.* limata*	VU	DD	VU
28	*A*.* minutula*		DD	UT
29	*A*.* minutoloides*		DD	UT
30	*A*.* mitis*	VU	DD	NT
31	*A*.* nigroaenea*		LC	UT
32	*A*.* nitida*		LC	UT
33	*A*.* nycthemera*	VU	DD	VU
34	*A*.* ovatula*		NT	LC
35	*A*.* pilipes*		LC	LC
36	*A*.* praecox*		LC	UT
37	*A*.* propinqua*		DD	DD
38	*A*.* ruficrus*		LC	VU
39	*A*.* subopaca*		LC	UT
40	*A*.* symphyti*	VU	DD	EN
41	*A*.* tibialis*		LC	LC
42	*A*.* vaga*		LC	UT
43	*A*.* varians*		LC	NT
44	*A*.* ventralis*		DD	UT
45	*A*.* viridescens*	VU	DD	VU
46	*A*.* wilkella*		DD	LC

The dominants were early spring mining bees with a wide environmental tolerance: eurytopic *Andrena haemorrhoa* (22.8% of the total abundance) and polytopic *A*.* ventralis* (16.6%), *A*.* vaga* (12.6%), *A*.* nigroaenea* (11.2%) and *A*.* cineraria* (12.7%) (Table 4). Two of the dominants, *Andrena ventralis* and *A*.* vaga*, are pollen specialists (oligolectic).

**Figure 3:**
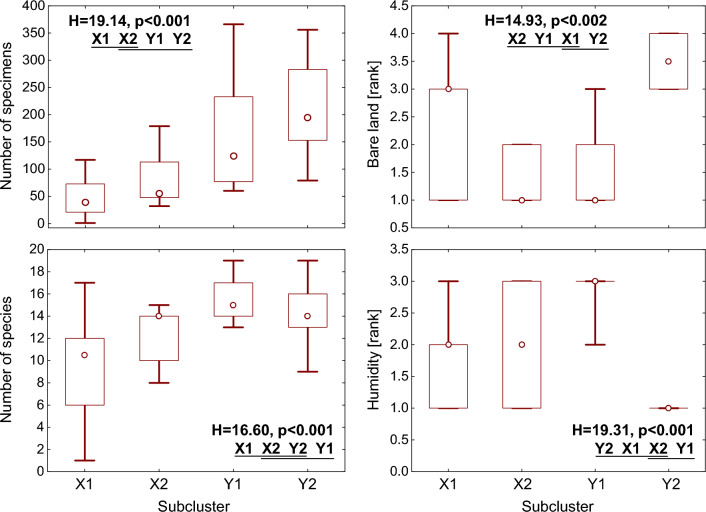
The species richness and abundance of mining bees, the availability of bare land and the soil humidity in SOM subclusters X1–Y2. Ranks used for bare ground: 1: ≤ 25%, 2: 26–50%, 3: 51–75%, and 4: > 75%. Ranks used for soil humidity: 1—autogenic (dry), 2—semi-hydrogenic, and, 3—hydrogenic (humid). Point—median, whiskers—inter-quartile range, H—Kruskal–Wallis test statistic (df = 3, N_X1_ = 18, N_X2_ = 5, N_Y1_ = 11, N_Y2_ = 6), which was used for inter-subcluster comparisons. The subclusters underlined by the same line were not found to be significantly different in post hoc comparisons.

The hierarchical cluster analysis identified two clusters of neurons (X and Y) in the output layer of the SOM (Fig. 2). Each cluster included two subclusters, which were ordered according to the gradients observed in the total abundance of mining bees: X1 (neurons A1–A3, B1–B3, C1–C4 and D1–D3) and X2 (neurons A4, A5, B4, B5) in cluster X, and Y1 (neurons C5, D4, D5, E3–E5 and F3–F5) and Y2 (neurons E1, E2, F1 and F2) in cluster Y (Figs. 2, 3).

Subcluster X1 contained only samples from open habitats, i.e. fallow lands, meadows, sandy grasslands and sand dune, which were mainly (> 80%) located on autogenic (dry) and semihydrogenic (semihumid) soil; however, this cluster also included one exception, i.e. from alder forest (Fig. 2, Table 3). Subcluster X2 grouped samples from mixed forests, fruit trees, a meadow and the abandoned farm (with willow species); these were situated on autogenic (dry), hydrogenic (wet) and semihydrogenic (semihumid) soils (Fig. 2, Table 3). Subcluster Y1 included only samples from open habitats (fallow lands and a meadow), on hydrogenic soil (with two exceptions), while all the samples in subcluster Y2 were collected from open habitats (fallow lands) on autogenic (dry) soil (Fig. 2, Table 3).

The abundance of mining bees increased through subsequent subclusters, i.e. from X1 to X2, Y1 and Y2, with a significant difference observed between subcluster X1 and subclusters Y1 and Y2 (Fig. 3). An upward trend was also observed in the first three subclusters (X1, X2 and Y1) in the species richness, with significant difference observed between X1 and Y1 (Fig. 3).

Significant differences were also observed in (1) the availability of bare ground (exposed surface) between X2, Y1 (lowest medians) and Y2 (highest median), and (2) the soil humidity between X1, Y2 (driest) and Y1 (most humid) (Fig. 3).

It should be noted that the highest values of species richness and abundance were observed both in subcluster Y2, characterised by low ground humidity (dry), and in subcluster Y1, with the highest median humidity (wet) (Fig. 3). Therefore, hypothesis (H1) “the mining bees demonstrate a narrow ecological amplitude” was not confirmed.

**Figure 4:**
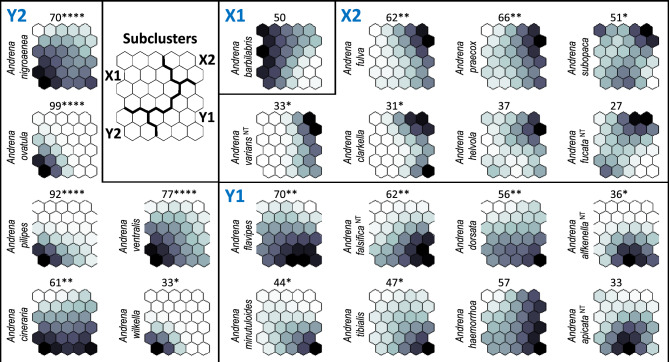
Mining bee species that were associated at *p* ≤ 0.1 with the self-organising map (SOM) subclusters X1, X2, Y1 and Y2. The shading was scaled independently for each species; the depth of the shading indicates the strength of the associations (based on virtual mining bee samples). Species with the same pattern occurred in similar habitats. The highest indicator value (IndVal; based on real mining bee samples) observed for a given species, and its significance level (**** *p* ≤ 0.0001; *** *p* ≤ 0.001; ** *p* ≤ 0.01; * *p* ≤ 0.05) are presented above the plane of each species. A smaller *p*-value indicates stronger evidence of association. Information in superscript next to the species name refers to the threat category (NT—near threatened); species without such information have lower threat categories.

Among the recorded 46 species, 17 (37%) were found to be indicators in terms of the Indicator Species Analysis, i.e. they exhibited significant (*p* ≤ 0.05) maximum IndVals. In subcluster X1 no species exhibited significant maximum IndVal. The numbers of indicator species were similar in the remaining three subclusters (X2, Y1 and Y2), i.e. five for X2, and six both for Y1 and Y2 (Fig. 4, Table 4); this resembles the trend observed for the species richness of mining bees (Fig. 3). As the result, hypothesis (H2) “there are no indicator species for particular habitats” was not confirmed.

Some species were recorded exclusively (but sporadically) in the samples assigned to the subclusters (those with A = 100% in Table 4). Of these, three species recorded only in X1 have an endangered status in Poland: *Andrena symphyti* (EN category), *A*.* gelriae* and *A*.* ruficrus* (both VU categories) (Tables 5, 6).

Five species were significantly associated with subcluster X2: *Andrena fulva*, *A*.* praecox*, *A*.* subopaca*, *A*.* varians* and *A*.* clarkella* (Fig. 4, Table 4). Two other species, *A*.* nycthemera* and *A*.* denticulata*, each represented by a single specimen, were recorded only in samples assigned to X2 (Table 4). Both species occur in early spring and summer. *Andrena nycthemera* has the threat category VU, and *A*.* denticulata* is regarded NT (Tables 5, 6).

The following six species were recorded to be indicators in cluster Y1:* Andrena tibialis*, *A*.* flavipes*, *A*.* dorsata, A*.* minutoloides*, *A*.* alfkenella* and *A*.* falsifica* (Fig. 4, Table 4). Two last species are near threatened (NT). Moreover, *Andrena alfkenella* and three other species were recorded only in samples assigned to Y1 (Tables 4, 5). Among them, *Andrena gallica* is critically endangered (CR), while *A*.* chrysopyga* and *A*.* limata* are regarded as vulnerable (VU) in Poland (Tables 5, 6).

Six species exhibited significant IndVals in subcluster Y2: *Andrena nigroaenea*, *A*.* ovatula*, *A*.* pilipes*, *A*.* ventralis*, *A*.* cineraria* and *A*.* wilkella* (Fig. 4, Table 4). The associations of *Andrena ovatula, A*.* nigroaenea, A*.* pilipes and A*.* ventralis* with Y2 were highly significant (*p* < 0.0001) (Fig. 4), and *A*.* wilkella* was recorded exclusively in the samples of this subcluster. None of the species was threatened (Table 6).

Out of the species found sporadically (at 1–2 sites), 67% belonged to the threat categories CR-NT (Tables 4, 5).

## Discussion

The 46 species recorded during this study represent approximately 10% of all bee species and about 46% of Polish andrenids (*Andrena* spp.), which currently constitute 100 species^4^. This latter proportion is similar to that recorded for other Aculeata taxa in the Kampinos National Park (*ca*. 50% pompilids, 46% chrysidids, 40% vespids)^39–41^. The number of mining bee species known currently to exist within the KNP is 59, as noted previously^10^ and in the present study. This value outnumbers almost all other national parks in Poland^10,62^. This richness of andrenid bee species can be attributed to the presence of diversified open areas mixed with various types of forests, and reflects the status of the KNP, together with its buffer zone, as one of the most important faunal refugia in the Polish lowlands^35^. This positive effect of the diversity of the KNP landscape has also been observed for other groups of Aculeata^39–41^.

Among the species observed in the KNP in the present study, *Andrena florivaga* was recorded for the first time in the Polish fauna^10^. Its presence might due to climate change, as the records in KNP are at the northernmost part of its range. Furthermore, the occurrence of *Andrena gallica* in Poland was also confirmed after more than 50 years, based on a specimen collected in KNP^34^.

The verified list of threatened *Andrena* species in Poland, based on Motyka^10^ and Wiśniowski et al.^34^, shows that 19 mining bee species (41%) recorded in the KNP are threatened, namely one species has the category CR (*Andrena gallica*), one species has the category EN (*A*.* symphyti)*, seven species are assigned as VU and 10 species as NT (Table 6). The percentage of endangered species (i.e. CR-NT) in the genus *Andrena* (41%) is much higher than those recorded in the other Aculeata studied in the KNP: e.g. only 13.6% of Pompilidae (out of 44 species previously identified in the KNP)^39,63^, 15.9% of Chrysididae^64^ (out of 44)^40^, and 4.5% of Vespidae^46^ (out of 22)^41^. This relative abundance of threatened species in the studied habitats highlights the role of the KNP in preserving mining bee species diversity and their conservation. All the noted dominant andrenid species are on the *European Red List of Bees* with LC and DD categories^29^. These dominants are early spring flying bees with a wide food and habitat tolerance; most are widespread in Poland and are often dominant in many habitats, including urban and suburban areas^20^. The exceptions are two pollen specialists, *Andrena ventralis* and *A*.* vaga*, foraging mainly on willow species (*Salix* spp.). Moreover, more than half of the oligolectic species found in KNP (*Andrena apicata, A*.* clarkella, A*.* denticulata, A*.* fulvago, A*.* gallica, A*.* gelriae, A*.* humilis, A*.* lapponica, A*.* mitis, A*.* nycthemera, A*.* praecox, A*.* ruficrus, A*.* symphyti, A*.* vaga, A*.* ventralis, A*.* viridescens*) specialize on *Salix* spp. Species that can use multiple resources are more likely to meet their resource needs in a greater diversity of habitats^65^, including anthropogenic ones^66^, whilst species with restricted diets may only meet their requirements in a limited subset of patches. The mosaic (of post-agriculture) habitats in KPN allows species to function, both those with broad tolerance and narrow habitat and food specializations. Willow thickets found in the KNP in or near the fallows and meadows, located on moist and moderately moist soils^35^, ensure the persistence of large populations of early spring andrenas^38^, including the oligolectic species^67^.

Among the oligolectic species, almost half are oligotopic, associated both with (1) open habitats such as fallows, grasslands, forest margins (*Andrena gallica*, *A*.* gelriae*, *A*.* mitis*)*,* flood-controlled sandbars, gravel pits (*A*.* nycthemera*), riverbanks, floodplains, river valleys, gravel pits (*A*.* symphyti*), (2) forested habitats: forests, forest edges, bogs (*A*.* ruficrus*, *A*.* lapponica*)^47^. The last species, *Andrena lapponica*, oligolectic on Ericaceae^47^, belongs to a group of species that in the KNP are relict of biocenoses from early post-glacial periods^38^. These species, with boreal-mountain distribution, occur mainly in very poor pine forests, with heather and blueberry. Plewka^38^ recommends that the KNP authorities should support these bees by clearing pine forests, especially on the southern slopes of the dunes, and not allowing heath and high and transitional bogs to overgrow. Michener^1^ and Westrich^68^ indicate that depending on the habitat, between 15 and 60% of the local bee species are strictly specialist (oligolectic) and collect pollen from only few plant species belonging to one plant family or genus. Specialist species must be able to find their specific plants in a complex environment. It is essential that food sources can be located to ensure reproductive fitness, because the availability of food is one of the major factors limiting the development of offspring and survival of adults^69^.

The highest species richness and abundance of andrenids were recorded in open habitats; the greatest abundance was noted in cluster Y2, which included only samples from fallow fields on autogenic (dry) soil, and the highest number of species was found in cluster Y1, containing samples from fallow fields and meadow, located mainly on hydrogenic (humid) soil (Figs. 2, 3). Earlier studies have shown that, for example, pompilids and chrysidids avoid wet habitats (open habitats on hydrogenic soils)^39,40^, but the highest abundance and richness of vespid wasps were recorded in open habitats on semihumid (semihydrogenic) soil^41^. Dry and warm areas are generally considered preferred habitats for most pollinators, especially bees, while wet marshy areas appear to be more species-poor^1^. However, some studies show that wet habitats are also important for bees. For example, Moroń et al.^14^ recorded 105 bee species, including 32 andrenid bees, on a wet/moist meadow in the Kraków-Wieluń Upland. The authors also found that the proportion of ground nesting bees and particular bee families (including Andrenidae) did not differ between study sites (wet/moist meadows) and xerothermic meadows in Ojców National Park, which resembles our results for subclusters Y1 (wet habitats) and Y2 (dry habitats) (Figs. 3, 4). Moreover, 35 andrenid species were noted in Ojców National Park on xerothermic grasslands, while 23 species were recorded in moist/wet (herbaceous) meadows in valleys (Wiśniowski *unpublished data*). Moroń et al.^14^ also showed that *Molinietum* meadows are characterised by diverse bee assemblages fauna with numerous rare and specialized species: these comprised 74% ground nesting species, 34% oligolectic, and 8% from the *Polish Red List*^32,33^. In the present study in the KNP, five rare species, i.e. with CR and VU threat categories, were associated with habitats located on moist and medium-humid soils (Tables 4, 5). It can be seen, therefore, that moist/wet habitats are very valuable for bees and are sometimes as species-rich as semi-natural grasslands. Moroń et al.^14^ emphasise the need to perform further investigations on the bee communities in wet habitats (wetlands and marshy areas) that belong to habitats that are particularly threatened by current climate change^70^. Unfortunately, wetlands are being degraded and lost due to pollution, overexploitation, climate change and human population growth. In recognition of these challenges, the RAMSAR Convention, an international treaty, was adopted in 1971 with the aim of addressing global concerns regarding wetland loss and degradation^71^.

Wet habitats are regarded as not very attractive to aculeates and are used more for foraging than for nesting^30^. However, to impede water exchange with the surrounding soil, the lipid cell linings made by most ground-nesting bees are hydrophobic. *Andrena* females impregnate the cell walls with two classes of chemicals secreted by the Dufour gland^72^. The water-repellent membrane protects the brood provision mass of pollen and nectar and the moisture-sensitive bee larva, allowing it to withstand even several hours of flooding^73^. Thus, the impregnated lining allows nesting in wetter soil^74^.

The differences in the habitats exploited by andrenids were reflected in the number of species exhibiting significant maximum IndVals in particular subclusters, i.e. their preference for respective environmental conditions. The number of such species may serve as a bio-indicator of the environment quality for a given group of animals^39–41,75^; this is supported by the fact that the number of the species with significant IndVals corresponded to the species richness of mining bees (Figs. 3, 4).

In subcluster X1, no species exhibited significant maximum IndVals.

Five indicator species for X2 (Fig. 4) have a wide ecological tolerance (Table 7). *Andrena fulva*, recorded in 35% of samples in the current study, has not been recorded in the KNP area previously^37^. It is a Western European species with a tendency to spread eastward. It is synanthropic, nests in clusters, and can establish colonies even in busy urban centres^76^.

**Table 7:** Ecological characteristics of *Andrena* species (according to Scheuchl and Willner^47^) with significant indicator values (IndVals; see Fig. 4); vertical distribution is marked according to the following symbols: #—occurrence from lowlands to uplands (0–800 m above sea level); ##—lowlands to mountane zone (0–1600 m above sea level); ###—lowlands to subalpine zone (0–2100 m above sea level); ####—lowlands to alpine zone (0-more than 2100 m above sea level).

Subcluster	No	*Andrena* species	Vertical distribution	Ecological tolerance			Preferred habitats												Nesting preferences/Soil moisture				Food preferences		On wing from			No. of generations
				Eurytopic	Polytopic	Oligotopic	Forests	Light forests	Forest edges	Clear cuts/clearings	Gardens, parks	Meadows	Embankments	Floodplains	Wastelands/fallow lands	Ruderal areas	Inland dunes/sandy areas	Sand pits/gravel pits	Dry	Dry and moderately moist	Moderately moist	Moderately moist and moist	Polylectic	Oligolectic	Early spring	Late spring	Summer	
**X2**	1	*A*.* fulva*	##		**x**			**x**	**x**		**x**		**x**							**x**			**x**		**x**			1
	2	*A*.* praecox*	##		**x**				**x**								**x**	**x**	**x**					**x** (*Salix*)	**x**			1(2)
	3	*A*.* subopaca*	###	**x**				**x**	**x**		**x**	**x**	**x**					**x**		**x**			**x**		**x**			2
	4	*A*.* varians*	##		**x**				**x**		**x**				**x**	**x**				**x**			**x**			**x**		1
	5	*A*.* clarkella*	###		**x**		**x**		**x**	**x**	**x**										**x**			**x** (*Salix*)	**x**			1
**Y1**	6	*A*.* flavipes*	##	**x**				**x**	**x**	**x**	**x**	**x**	**x**			**x**		**x**		**x**			**x**		**x**			2
	7	*A*.* falsifica*	##		**x**			**x**	**x**			**x**			**x**	**x**				**x**			**x**		**x**			1
	8	*A*. *dorsata*	##		**x**				**x**	**x**		**x**	**x**					**x**		**x**			**x**		**x**			2
	9	*A*.* alfkenella*	##		**x**				**x**			**x**				**x**				**x**			**x**		**x**			2
	10	*A*.* minutoloides*	##	**x**				**x**	**x**	**x**	**x**	**x**	**x**			**x**		**x**	**x**				**x**		**x**			2
	11	*A*.* tibialis*	##		**x**				**x**		**x**	**x**						**x**		**x**			**x**		**x**			2
**Y2**	12	*A*.* nigroaenea*	###		**x**				**x**		**x**	**x**	**x**					**x**				**x**	**x**		**x**			1(2)
	13	*A*.* ovatula*	###		**x**						**x**	**x**	**x**			**x**		**x**		**x**			**x**			**x**		2
	14	*A*.* pilipes*	##			**x**									**x**	**x**	**x**		**x**				**x**		**x**			2
	15	*A*.* ventralis*	##		**x**								**x**	**x**				**x**				**x**		**x** (*Salix*)	**x**			1
	16	*A*.* cineraria*	####		**x**				**x**		**x**	**x**	**x**			**x**		**x**		**x**			**x**		**x**			(1)2
	17	*A*.* wilkella*	###		**x**				**x**			**x**				**x**		**x**		**x**				**x** (Fabaceae)		**x**		(1)2

Six indicator species for Y1 (Fig. 4) have a wide environmental tolerance; they occur from lowlands to mountains, apart from the subalpine and alpine zones (Table 7). According to literature^43,45,47,48^, all are polylectic, early spring species, of which five prefer dry or moderately moist habitats, and *Andrena minutuloides* prefers dry habitats (Table 7). However, our present data indicates that *Andrena alfkenella* and *A*.* minutuloides* are significantly associated with Y1, resulting from their presence at humid sites assigned to this subcluster. This indicates a different habitat preference than reported in the literature. Therefore, hypothesis (H3) “the studied mining bees have the same ecological preferences to those presented in the literature” was not confirmed.

Six indicator species for Y2 (Fig. 4) avoid forests (Table 7). All but one are polytopic, preferring sites of different soil humidity (Table 7). *Andrena nigroaenea* and *A*.* ventralis* are reported in the literature^43,45,47,48^ as being associated with moist habitats (Table 7), while our present data suggest they are highly significant indicators for the typically “dry” subcluster Y2 (Fig. 4); this contradicts hypothesis H3.

While insects are declining in many parts of the world, they constitute only 8% of the assessed species in the IUCN Red List. A key role in safeguarding many insect species could be played by protected areas^77,78^. In Poland, protected areas, especially national parks, are very important refuges for andrenid bees^10,62^. In the Kampinos National Park, 59 species noted in the present and previous studies^10^ have been recorded, i.e. 85% of the taxa from the region. Nationwide, 86 of the 95 *Andrena* bee species (90%) known in Poland were identified in Polish national parks^10,62^.

There are still many gaps in knowledge about the species diversity of wild bees in key regions of the world, including Europe, especially its southern and eastern parts^79^. Current data suggests that fewer wild bee species are present in Poland than in neighbouring Germany, Czech Republic, Slovakia or Ukraine^80^; however, this number will probably increase with further intensive research (e.g.^81,82^). This also applies to some challenging bee groups, such as the genus *Andrena* (e.g.^83,84^). It should be stressed that more than 55% of all European known species of bees were described as ‘Data Deficient’ in the first, and only, IUCN Red List for the continent^29^. These understudied species should be prioritised in future sampling programmes, and in general, more taxonomic work is needed to provide a better understanding of their ecology, biogeography and conservation status^80^.

## Conclusions

1. The studied mining bees were found to demonstrate a wide ecological amplitude. Habitats located in dry and wet soils were characterised by both high abundance and substantial species richness; however, generally speaking, dry and warm areas are considered to be preferred by most pollinators, while wet marshy areas are comparatively poor in species. As a result, hypothesis H1 was not confirmed.
2. Seventeen (37%) species were found to be indicators. They exhibited a significant preference for specific habitat conditions. Therefore, the hypothesis H2 was not confirmed.
3. The indicator species were distinguished in subclusters with sites located on autogenic (dry) and hydrogenic (humid) soils; this also contradicts hypotheses H1 and H2.
4. Compared to previous studies, *Andrena nigroaenea* and *A*.* ventralis* showed a greater preference for soil with lower humidity, while *A*.* alfkenella* and *A*.* minutuloides* preferred habitats with higher humidity. As a result, the hypothesis H3 was not confirmed.
5. Among the recorded 46 species, 19 (41%) were assigned to the CR-NT threat categories. Such a strong presence of threatened species in the KNP indicates the high quality of the studied habitats, and confirms the role of national parks in conserving mining bee species diversity.
6. The genus *Andrena* demonstrated a much higher percentage of endangered species than those recorded in the other taxa of Aculeata studied in Kampinos National Park.

### Supplementary Information

Supplementary Information.
