# Supplementary material for: Ecological amplitude and indication potential of mining bees (Andrena spp.): a case study from the post-agricultural area of the Kampinos National Park (Poland)
Source: Sci Rep. 2024 Apr 28;14:9738. doi: 10.1038/s41598-024-59138-9 (PMC11056373; doi:10.1038/s41598-024-59138-9)
Supplement: Supplementary file 1 — Supplementary Information. [file 41598_2024_59138_MOESM1_ESM.pdf]

proba	Annigr	Anbico	Anbarb	Anfulv	Anprae	Ansubo	Anovat	Angelr	Anvari	Anclar
1FT02	2	0	6	12	4	2	0	0	0	0
2SG02	0	0	7	0	0	3	0	0	0	0
3SD02	0	0	17	0	0	3	0	0	0	0
4ME02	2	0	3	6	2	0	0	0	0	0
5SG02	1	0	2	4	1	0	0	0	0	0
6AF02	2	0	0	10	16	2	0	0	1	1
7FR02	1	0	1	8	1	5	0	0	1	0
7FR03	0	0	0	0	2	11	0	0	0	2
8AF02	0	0	3	0	0	4	0	0	0	0
9FA02	0	0	1	0	0	0	0	0	0	0
9FA03	13	0	1	0	2	0	0	0	0	0
9FA04	14	0	0	1	0	5	0	0	0	0
9FA05	17	0	2	0	2	1	0	0	0	0
10FA03	48	0	1	0	0	2	6	0	0	0
10FA04	54	0	7	0	0	1	1	0	0	0
11FA03	8	0	4	0	0	0	0	0	0	0
11FA04	10	0	4	0	0	2	0	0	0	0
11FA05	12	0	1	0	1	6	0	0	0	0
12FA03	9	0	1	0	0	0	0	0	0	0
12FA04	8	0	2	0	0	2	0	0	0	0
13FA03	6	0	13	0	0	0	0	0	0	0
13FA04	19	0	33	0	0	0	0	0	0	0
14FA03	15	0	2	2	4	0	0	0	0	2
14FA04	17	0	0	2	3	0	0	0	0	0
15ME03	0	0	1	0	0	0	0	0	0	0
15ME04	14	0	1	0	1	1	0	0	0	0
16FA04	8	1	0	0	0	0	0	0	0	0
16FA05	1	0	0	3	3	0	0	0	0	0
16FA06	12	0	0	2	0	0	0	0	0	0
17FA04	3	0	0	0	3	0	0	0	0	0
18FA04	0	0	0	0	1	0	0	0	0	0
18FA05	12	0	1	3	1	0	0	0	1	0
19FA04	0	0	1	0	0	0	0	0	0	0
20FA05	40	0	6	0	0	0	5	0	0	0
21FA05	11	0	4	0	0	0	4	0	0	0
21FA06	24	0	26	0	0	0	1	0	0	0
22FA05	64	0	2	2	0	0	10	0	0	0
22FA06	5	0	5	2	1	0	0	2	0	0
23FA05	28	0	3	0	4	0	5	0	0	0
23FA06	5	0	7	4	1	0	0	0	0	0

proba	Anhelv	Anfuca	Anpili	Anvent	Anflav	Anfals	Andors	Analfk	Ancine	Anwilk
1FT02	12	0	0	14	2	0	0	0	1	0
2SG02	0	0	0	1	1	0	0	0	0	0
3SD02	2	0	0	3	1	0	0	0	0	0
4ME02	0	0	0	2	0	0	0	0	1	0
5SG02	0	0	0	1	5	0	0	0	0	0
6AF02	1	0	0	0	0	0	0	0	5	0
7FR02	1	2	0	0	0	0	1	0	3	0
7FR03	0	7	0	0	0	0	0	0	0	0
8AF02	0	0	0	0	0	0	0	0	3	0
9FA02	0	0	0	0	0	0	0	0	0	0
9FA03	0	0	0	13	7	0	0	0	1	0
9FA04	0	0	3	13	48	1	1	0	20	0
9FA05	1	0	0	2	8	0	1	0	17	0
10FA03	0	0	4	53	3	0	1	0	73	2
10FA04	6	1	5	151	4	0	0	0	80	1
11FA03	0	1	0	11	0	0	0	0	1	0
11FA04	0	0	0	15	4	0	1	0	18	0
11FA05	0	0	0	9	0	0	0	0	8	0
12FA03	0	0	0	4	2	2	1	0	1	0
12FA04	0	0	0	26	3	1	4	0	6	0
13FA03	0	0	0	3	0	0	0	0	3	0
13FA04	0	0	0	29	2	0	2	0	9	0
14FA03	2	0	0	6	5	0	3	0	38	0
14FA04	0	0	0	8	3	4	10	1	72	0
15ME03	0	0	0	1	0	0	0	0	0	0
15ME04	0	0	0	4	6	0	0	2	40	0
16FA04	0	0	0	2	1	0	0	0	3	0
16FA05	0	0	0	2	1	1	1	0	9	0
16FA06	1	0	0	5	2	2	1	0	11	0
17FA04	0	0	0	0	3	1	1	0	4	0
18FA04	0	0	1	9	21	5	0	1	5	0
18FA05	0	0	0	4	35	11	1	0	9	0
19FA04	0	0	0	8	8	1	5	3	3	0
20FA05	0	0	6	59	2	2	2	0	22	0
21FA05	0	0	2	42	0	0	0	0	9	0
21FA06	0	0	0	40	2	0	0	0	3	0
22FA05	0	3	4	45	4	2	0	0	52	0
22FA06	4	0	0	25	0	0	0	0	6	0
23FA05	0	0	3	75	4	0	1	0	10	0
23FA06	2	2	0	35	4	0	0	0	5	0

proba	Anmdes	Antibi	Anhaem	Anapic	Anbima	Anchry	Anchpy	Andent	Anflor	Anfugo
1FT02	0	0	107	0	8	0	0	0	0	0
2SG02	0	0	0	0	0	0	0	0	0	0
3SD02	0	0	0	1	0	0	0	0	0	0
4ME02	0	1	4	0	0	0	0	0	0	0
5SG02	0	0	4	0	0	0	0	0	0	0
6AF02	0	0	14	0	0	2	0	0	0	0
7FR02	0	0	24	0	0	0	0	0	0	0
7FR03	0	0	23	0	0	0	0	1	0	0
8AF02	0	0	6	0	0	0	0	0	0	0
9FA02	0	0	0	0	0	0	0	0	0	0
9FA03	2	0	5	0	0	0	0	0	0	1
9FA04	0	2	5	3	0	1	0	0	1	0
9FA05	0	1	8	0	0	2	0	0	0	0
10FA03	0	0	0	0	4	0	0	0	0	1
10FA04	0	0	10	0	10	0	0	0	0	2
11FA03	0	0	0	0	0	0	0	0	0	0
11FA04	0	0	2	0	0	0	0	0	0	4
11FA05	0	2	11	1	0	0	0	0	2	0
12FA03	0	0	6	1	0	0	0	0	0	0
12FA04	0	0	6	3	0	0	0	0	2	0
13FA03	0	0	0	0	0	0	0	0	0	1
13FA04	0	0	7	0	0	0	0	0	0	1
14FA03	16	4	69	0	0	0	0	0	0	0
14FA04	2	12	213	0	1	0	0	0	0	0
15ME03	0	0	0	0	0	0	0	0	1	0
15ME04	0	2	12	2	0	1	0	0	0	0
16FA04	0	2	4	0	0	0	0	0	1	0
16FA05	0	1	73	0	0	0	1	0	0	0
16FA06	0	4	243	0	0	0	0	0	0	0
17FA04	0	0	24	3	0	0	0	0	0	0
18FA04	4	2	10	0	0	0	0	0	1	0
18FA05	2	0	37	0	0	5	0	0	0	0
19FA04	7	0	3	3	1	0	0	0	0	0
20FA05	0	2	14	1	0	0	0	0	0	0
21FA05	0	0	3	0	0	1	0	0	0	0
21FA06	0	0	2	0	0	0	0	0	0	0
22FA05	0	0	15	1	0	0	0	0	0	0
22FA06	0	0	9	0	0	0	0	0	0	0
23FA05	0	0	11	0	0	0	0	0	0	0
23FA06	0	1	4	0	0	0	0	0	0	0

proba	Anfuda	Angali	Angrav	Anhumi	Anlabi	Anlapo	Anlima	Anminu	Anmiti	Anniti
1FT02	0	0	0	0	0	0	0	2	3	1
2SG02	1	0	0	0	1	0	0	0	0	0
3SD02	0	0	0	0	0	4	0	0	0	0
4ME02	1	0	0	0	0	0	0	0	0	0
5SG02	0	0	0	0	0	0	0	0	0	0
6AF02	0	0	0	0	0	0	0	0	3	1
7FR02	0	0	0	0	0	5	0	0	0	1
7FR03	0	0	0	0	0	1	0	0	0	0
8AF02	1	0	0	0	0	0	0	0	0	0
9FA02	0	0	0	0	0	0	0	0	0	0
9FA03	0	0	0	0	0	0	0	0	0	1
9FA04	0	1	1	0	0	0	0	0	1	1
9FA05	0	0	0	0	0	0	0	0	0	0
10FA03	0	0	0	0	0	0	0	0	0	0
10FA04	0	0	1	0	0	0	0	4	1	5
11FA03	0	0	0	0	1	0	0	2	2	1
11FA04	0	0	0	0	7	0	0	0	0	1
11FA05	0	0	0	0	1	0	0	0	1	1
12FA03	0	0	0	0	0	0	0	0	0	0
12FA04	0	0	0	0	0	0	0	0	0	3
13FA03	0	0	0	0	0	0	0	0	0	0
13FA04	0	0	0	0	2	0	0	0	0	3
14FA03	0	0	0	0	1	0	0	0	2	1
14FA04	0	0	0	1	0	0	0	0	1	3
15ME03	0	0	0	0	0	0	0	0	0	1
15ME04	0	0	0	0	2	0	0	0	0	13
16FA04	0	0	0	0	2	0	0	2	0	14
16FA05	0	0	1	0	0	0	0	0	0	1
16FA06	0	0	0	0	4	0	0	0	0	3
17FA04	0	0	1	0	1	0	1	0	0	1
18FA04	0	0	1	0	6	0	0	0	0	4
18FA05	0	0	0	0	0	0	0	0	1	2
19FA04	0	0	0	0	3	0	0	1	0	1
20FA05	0	0	0	0	0	2	0	0	1	2
21FA05	0	0	0	0	0	0	0	0	0	3
21FA06	0	0	0	0	1	0	0	0	0	0
22FA05	0	0	0	0	0	0	0	0	1	6
22FA06	0	0	0	0	0	0	0	0	0	0
23FA05	0	0	0	0	1	0	0	0	0	2
23FA06	0	0	0	2	1	3	0	0	0	1

proba	Annyct	Anprop	Anrufi	Ansypm	Anvaga	Anviri
1FT02	0	0	0	0	3	0
2SG02	0	0	0	0	0	0
3SD02	0	0	0	0	1	0
4ME02	0	0	0	0	10	0
5SG02	0	0	0	0	3	0
6AF02	1	0	0	0	54	0
7FR02	0	0	0	0	1	0
7FR03	0	0	0	0	1	0
8AF02	0	0	0	0	1	0
9FA02	0	0	0	0	0	0
9FA03	0	0	0	0	7	0
9FA04	0	0	0	0	80	0
9FA05	0	0	1	0	10	0
10FA03	0	0	0	0	85	0
10FA04	0	0	0	0	12	0
11FA03	0	0	0	0	3	0
11FA04	0	0	0	0	13	0
11FA05	0	0	0	0	7	0
12FA03	0	0	1	0	5	0
12FA04	0	0	0	0	17	0
13FA03	0	0	0	0	3	0
13FA04	0	0	0	0	10	0
14FA03	0	0	0	0	61	0
14FA04	0	0	0	0	13	0
15ME03	0	0	0	0	0	0
15ME04	0	0	0	0	23	0
16FA04	0	1	0	0	1	1
16FA05	0	0	0	0	2	0
16FA06	0	0	0	0	4	2
17FA04	0	0	0	0	13	1
18FA04	0	0	0	0	6	0
18FA05	0	0	0	0	46	0
19FA04	0	1	0	0	13	1
20FA05	0	0	0	0	5	0
21FA05	0	0	0	0	0	0
21FA06	0	0	0	0	4	0
22FA05	0	0	0	0	7	0
22FA06	0	0	0	0	8	1
23FA05	0	0	0	0	6	0
23FA06	0	0	0	1	8	0
